# Is There ANA Risk? A Retrospective Analysis Assessing the Long-Term Psychiatric Outcomes in Patients Testing Positive for Anti-Nuclear Antibodies, in the Absence of an Autoimmune Disease diagnosis

**DOI:** 10.1192/bjo.2025.10098

**Published:** 2025-06-20

**Authors:** Katharine Lynch-Kelly, Danish Hafeez, Vasishta Polisetty, Katharina Schmack, Thomas Pollak

**Affiliations:** ^1^IOPPN, King’s College London, London, United Kingdom; ^2^Neural Circuits and Immunity in Psychosis Laboratory, Francis Crick Institute, London, United Kingdom

## Abstract

**Aims:** Antinuclear antibody (ANA) is a sensitive but non-specific blood test frequently undertaken as part of the clinical assessment for a number of autoimmune diseases. While ANA positivity is associated with a number of autoimmune diseases, such as systemic lupus erythematosus (SLE), approximately one fifth of the population will test positive without having or subsequently developing an autoimmune disease. While there is a growing body of evidence demonstrating that patients with an autoimmune disease are more likely to develop psychiatric disorders, such as schizophrenia, the risk in patients who test positive for ANA but who never develop an autoimmune disease has not been established.

**Methods:** We undertook a retrospective cohort analysis using TriNetX, a large real-world population database, consisting of anonymised health records of over 250 million patients across 19 countries. Patients aged 16–90 years, without a recorded ICD diagnosis of an autoimmune disease were identified and divided into two cohorts – those with at least one positive ANA blood test, matched against those with at least one negative ANA blood test in the absence of any positive ANA antibody results. Confounding risk factors were controlled through propensity score matching for age, sex, sociodemographics, clinical characteristics and psychotropic medication use. Primary outcome was the incidence of and hazard ratios for psychiatric diagnoses from 3 months–10 years after the ANA test result.

**Results:** 454,740 patients were included in the primary analysis, 227,370 in the ANA positive group, 227,370 in the ANA negative group. There was no statistically significant difference in the risk of diagnosis of overall F20–29 diagnosis (HR 0.939, p=0.0674) and specifically F20 Schizophrenia (HR 0.964, p=0.5870).

**Conclusion:** A positive ANA blood test in the absence of an autoimmune disease was not associated with an increased long-term risk of psychiatric disorders. This result suggests that clinical testing of ANA in patients presenting with psychiatric disorders without features suggestive of a systemic autoimmune disease may be unwarranted.

